# Jewish Contributions to Plastic and Reconstructive Surgery

**DOI:** 10.5041/RMMJ.10577

**Published:** 2026-04-26

**Authors:** Daniel M. Portnoy, Coral Levkovitz, Zachary M. Stauber, Nicholas A. Mirsky, Seth R. Thaller

**Affiliations:** 1 University of Miami Miller School of Medicine, Miami, FL, USA; 2 Florida International University Herbert Wertheim College of Medicine, Miami, FL, USA; 3 DeWitt Daughtry Family Department of Surgery, University of Miami Miller School of Medicine, Miami, FL, USA

**Keywords:** History of medicine, Jews, physicians, plastic surgery, reconstructive surgical procedures

## Abstract

While individual Jewish plastic surgeons have been recognized in the literature for their groundbreaking achievements, a comprehensive review of their collective contributions to plastic surgery is lacking. The goal of this review is to document and highlight the major advancements made by Jewish physicians whose work significantly impacted plastic and reconstructive surgery from its inception as a discipline to the present day. A comprehensive literature search was conducted to identify key developments in the history of plastic surgery, which were then narrowed to those made by Jewish practitioners. The Jewish identity of these practitioners, along with the significance of their work, was verified through historical records, newspaper archives, and peer-reviewed publications. Contributions were then organized chronologically to construct a cohesive historical narrative. Beginning in the 1800s and accelerating through the 20th century, Jewish plastic surgeons have been instrumental in shaping the field. Their innovations include advancements in the treatment of facial deformities, the development of the lumpectomy with adjunctive radiation therapy as a less invasive alternative to radical mastectomy, and the founding of key institutions such as the American Society of Plastic Surgeons and the International College of Surgeons. Additional breakthroughs include the use of albumin in burn management, foundational work in angiogenesis physiology, and numerous innovations in cosmetic surgery. The history of plastic and reconstructive surgery is a rich tapestry of discovery and evolution. Fully understanding its development is not possible without acknowledging the enduring and transformative contributions of Jewish surgeons and researchers.

## INTRODUCTION

Plastic surgery began to emerge as a formal specialty in the mid-1800s. However, evidence of reconstructive surgical practices dates back much further. Ancient Egyptian texts, including the *Edwin Smith Surgical Papyrus*—written over 2,000 years ago—describe primitive techniques for treating mandibular and nasal fractures.^1^ Similarly, documentation from India as early as 600 BCE details the use of cutaneous flaps for nasal reconstruction.^2^ For centuries, physicians around the world have employed flaps, grafts, and various other techniques to reconstruct parts of the human body, particularly the face.^3^–^6^

“Plastic” in the context of surgery first appeared in 1818, when Karl Ferdinand von Graefe published his seminal work *Rhinoplastik*, which outlined techniques for nasal reconstruction.^7^ Two decades later, in 1838, Eduard Zeis formally established plastic surgery as a recognized surgical specialty.^8^ Since then, the field has evolved significantly—from advancements in bony reconstruction and the introduction of skin grafting in the 1860s, to the rise of cosmetic surgery in the 1890s.^1^

This specialty experienced a dramatic surge in innovation during World War I, spurred by the need to address the gruesome facial injuries soldiers sustained on the battlefield. Physicians responded with creative techniques to repair lacerations, burns, and disfigurements. These wartime innovations laid the groundwork for modern approaches. They have since been refined to improve healing, safety, and aesthetic outcomes in plastic and reconstructive surgery.^3^ Yet, the remarkable progress of plastic surgery over the past two centuries would not have been possible without the contributions of notable Jewish surgeons. Since the mid-1800s, Jewish physicians have been instrumental in advancing the field by establishing national societies, pioneering novel surgical techniques, and promoting discourse to shape the future of the specialty. Their impact on plastic and reconstructive surgery is substantial and warrants formal recognition.

This historical review explores the influence of these pioneering Jewish surgeons and acknowledges their contributions to shaping the modern landscape of plastic surgery. The perception of plastic surgery within Judaism remains complex. Procedures that promote healing or restore function are generally accepted within Jewish ethical thought, while surgeries performed solely for aesthetic enhancement may be seen by some as conflicting with core Jewish values. Understanding both the historical contributions and cultural context of Jewish involvement in plastic surgery provides a more comprehensive view of the specialty’s evolution and enduring impact.

Ethical perspectives within Judaism have historically shaped the acceptance of surgical intervention, including procedures that overlap with modern plastic and reconstructive surgery. Jewish medical ethics broadly permit surgical procedures when performed to restore function, preserve health, alleviate physical or psychological suffering, protect human dignity, or enable an individual to maintain livelihood or social well-being. In contrast, interventions pursued solely for vanity, without therapeutic or restorative purpose, have traditionally been viewed with greater ethical hesitation. This ethical framework contextualizes Jewish physicians’ historical engagement in reconstructive, restorative, and function-preserving surgical innovation, while acknowledging the nuanced perspectives surrounding aesthetic enhancement.

Numerous additional Jewish contributors, particularly in Europe, Israel, and the United Kingdom, played important roles in the development of plastic and reconstructive surgery in their communities, but are beyond the scope of this representative historical review.

## THE BEGINNINGS OF PLASTIC SURGERY

### Dr Julius Wolff (1836–1902)

The foundations of plastic surgery as a formal specialty are closely intertwined with the development of orthopedic surgery. In Germany, Jewish surgeon Julius Wolff emerged as a pioneering figure in the mid-1800s. He was instrumental in establishing orthopedics as a distinct medical discipline and introduced numerous innovative surgical techniques to correct bone and joint deformities. Among his most influential contributions was the formulation of “Wolff’s Law,” which proposed that the trabecular structure of bone adapts in response to mechanical loading.^9^ This groundbreaking insight was pivotal in advancing the understanding of bony architecture and its adaptive processes. Wolff’s work laid the foundation for modern concepts of bone injury, self-repair, and, ultimately, the evolution of reconstructive surgery.

### Dr Jacques Joseph (1865–1934)

German surgeon Jacques Joseph received four years of orthopedic surgical training under the mentorship of Julius Wolff at the University Polyclinic in Berlin, Germany. However, their relationship ended abruptly in 1896 after Joseph successfully corrected a young boy’s prominent ear deformity. Despite the operation’s success, Wolff disapproved of the perceived risk involved and subsequently removed Joseph from the training program.^10^

News of Joseph’s surgical accomplishment quickly spread. The next major milestone in his career came with the correction of a nasal deformity in an adult male patient. Joseph recognized the profound psychological impact of facial deformities and the human desire for social acceptance—a concept he referred to as “anti-dysplasia.”^11^ As his reputation grew, he became widely respected within the emerging field of aesthetic surgery. He is now considered the father of modern aesthetic medicine.

Throughout his career, Joseph developed groundbreaking techniques for reconstructing facial deformities, particularly those involving the nose. He pioneered the use of skin grafts and flaps and is credited with developing the first modern rhinoplasty procedures.^10^ In his landmark paper, *Nasal Reductions*, he described the use of osteotomies and cartilage modifications to reshape the nasal structure. For example, he explained that he would reduce the size of the nasal bones by sawing through the frontal process of the upper jaw and repositioning the nasal sidewall closer to the midline to achieve a more refined appearance.^12^ Joseph consistently emphasized surgical precision to ensure optimal functional and cosmetic outcomes for his patients.

### Dr Joseph Safian (1886–1993)

Joseph Safian, a direct disciple of Jacques Joseph, played a pivotal role in transmitting Joseph’s aesthetic and reconstructive principles to the United States. After emigrating from Europe, Safian became a prominent rhinoplasty surgeon in New York City, where he advanced Joseph’s techniques through clinical practice, teaching, and extensive lecturing. He emphasized meticulous preoperative analysis and individualized surgical planning, helping to establish enduring standards in nasal and facial aesthetic surgery.^13^

## THE AMERICAN SOCIETY OF PLASTIC SURGEONS

Jewish physicians in the USA continued to make significant contributions to the advancement of plastic surgery. At the time, the American Society of Oral Surgeons had recently formed but restricted membership to individuals holding both medical and dental degrees. Despite differing personalities, Dr Jacques Maliniac and Dr Gustave Aufricht collaborated to establish the American Society of Plastic Surgeons (ASPS), which broadened access to those focused exclusively on plastic and reconstructive surgery.^14^ As post-war emigrants from Europe, both Maliniac and Aufricht were visionary trailblazers whose legacy shaped the future of American plastic surgery. Today, the ASPS is the largest organization of Board-certified plastic surgeons in the world.

### Dr Jacques Maliniac (1889–1976)

Jacques Maliniac was born in Warsaw, Poland. He developed an interest in plastic surgery during his service as a medical doctor in the Russian Army during World War I. There, he witnessed the devastating physical trauma sustained by soldiers, which inspired his dedication to treating patients with severe injuries.^15^ In 1923, Maliniac traveled to the United States and, recognizing the rising antisemitism in Europe, chose to remain in New York City, where he opened a private practice.

While practicing in New York, Maliniac played a pivotal role in establishing a plastic surgery division within the Department of Hospitals in New York City. He went on to create the first dedicated Plastic Surgery Service in a municipal hospital at City Hospital on Roosevelt Island. He contributed to the refinement of various surgical techniques, particularly in breast reconstruction. Maliniac challenged existing anatomical assumptions and emphasized the importance of precise incision placement, thoughtful flap design, and the maintenance of adequate blood supply throughout the procedure.^16^

### Dr Gustave Aufricht (1895–1980)

The same year, 1923, Gustave Aufricht was invited to New York to establish a plastic surgery division at a small private hospital. Before emigrating, Aufricht had served on the Hungarian side during World War I, treating injured soldiers. He had also trained under the renowned Jacques Joseph, a pioneer in modern rhinoplasty.^10^ Although his initial plastic surgery division in New York did not materialize as planned, Aufricht chose to remain in the USA, motivated by antisemitic concerns similar to those of Maliniac.

Aufricht’s move to New York facilitated his connection with Maliniac, Dr Lyndon Peer, Dr Clarence Straatsma, and other formative members of what would become the ASPS.

## A REVOLUTION IN BURN TREATMENT

### Dr Isidore Ravdin (1894–1972)

Isidore Ravdin was another prominent Jewish American surgeon who played a crucial role in advancing the treatment of war casualties. He graduated from the University of Pennsylvania Medical School in 1918. Later, he would serve as surgeon-in-chief at the Hospital of the University of Pennsylvania (HUP) and the John Rhea Barton Professor of Surgery.^17^ Over the course of his career, he held numerous academic and clinical roles at the institution, including surgical chief resident, associate, instructor in surgery, and eventually chair of surgical research. Ravdin’s research interests spanned various organ systems, particularly the gallbladder and liver. However, he is best known for his pioneering work on blood substitutes, especially the use of albumin in the treatment of shock victims during wartime.^17^ He was a strong advocate for increased medical attention in veteran and military hospitals, arguing that general surgery residents should receive part of their training in these settings. While some criticized military hospitals for having a limited focus on war-related injuries, Ravdin firmly rejected this notion. He believed that these institutions offered a comprehensive training environment and that their integration into residency programs would benefit both future surgeons and the military community.^18^

Following the attack on Pearl Harbor in 1942, Ravdin collaborated with the National Research Council and the American Red Cross to explore the use of albumin injections to treat burn victims suffering from plasma loss. He and his team administered this therapy to seven patients, all of whom demonstrated successful recoveries marked by edema resolution.^19^ Albumin proved to be a highly effective treatment due to its ability to quickly restore circulating blood volume and its greater availability compared to plasma. Ravdin’s work had a transformative impact on the clinical management of burn injuries, not only for US military personnel but for patients worldwide suffering from burns, shock, or hemorrhage.

### Dr Leo Aryeh Bornstein (1922–1980)

Leo Aryeh Bornstein, a Polish-American immigrant known as one of the forefathers of plastic surgery in Israel, made important contributions to the management of severe burn injuries during a formative period in the evolution of modern burn care. He graduated from the Royal College of Surgeons in Edinburgh, Scotland, and did further training in England before joining the US army in 1943. Following his experiences in World War II, Bornstein decided to move to what would eventually become the state of Israel. In 1949, he briefly moved to New York to complete a plastic surgery fellowship at Harlem Hospital, only to return to Tel Aviv one year later and establish the country’s first plastic surgery department. His work, largely based on his experiences at the Burn Infirmary in Manchester and US Army hospitals, emphasized early wound management, physiological stabilization, and reconstructive planning, complementing contemporaneous advances in burn physiology and wound healing. These foundational principles underpin the multidisciplinary framework that guides modern burn reconstruction.^20^

## PIONEER OF BODY-CONTOURING SURGERY AND FOUNDER OF THE INTERNATIONAL COLLEGE OF SURGEONS

### Dr Max Thorek (1880–1960)

In 1935, Max Thorek founded the International College of Surgeons (ICS), an organization dedicated to advancing surgical education and global collaboration. Originally from Hungary, Thorek immigrated to Chicago with his family to escape antisemitic violence after the murder of his brother in 1897. He went on to attend Rush Medical College, in Chicago, Illinois. Later, he became the founder and surgeon-in-chief of the American Hospital, now known as Thorek Memorial Hospital, also in Chicago.^21^

Thorek made notable contributions to both aesthetic and general surgery. In the realm of cosmetic procedures, he introduced an early method for mammoplasties and abdominoplasties that helped shape later approaches to body-contouring surgeries. For mammoplasty, he pioneered the free transplantation of the nipple—a technique in which the nipple–areola complex is completely removed and then repositioned following breast reshaping to ensure symmetry and aesthetic outcomes.^22^ In abdominoplasty, he strategically placed incisions in less visible areas to minimize scarring and employed suturing techniques to reduce skin tension and promote optimal wound healing.^22^

Beyond aesthetic surgery, Thorek also made significant contributions to general surgery. He helped develop and popularize techniques such as partial hepatectomy for patients with gallbladder cancer, along with various improvements in gallbladder surgery. These advancements underscored his versatility and lasting impact across multiple surgical disciplines.^23^

Perhaps Thorek’s most enduring legacy is the establishment of the International College of Surgeons (ICS). As tensions rose on the brink of World War II, Thorek envisioned a global forum that would transcend political divisions and foster collaboration among surgeons worldwide. That vision led to the founding of the ICS. Today it includes several thousand members from more than 100 countries. The organization continues to advance the field of surgery through education, research, and humanitarian missions.

## JEWISH PIONEERS OF MODERN AESTHETIC AND RECONSTRUCTIVE SURGERY

### Dr Bernard L. Kaye (1927–2008)

Bernard L. Kaye was a key figure in the founding of the American Society for Aesthetic Plastic Surgery (ASAPS) in 1967. Later Kaye served as its president in 1980.^24^ Born in 1927, Kaye earned both his medical degree and a degree from the Harvard School of Dental Medicine, in Boston, Massachusetts, in 1953. He went on to complete a general surgery residency at Yale University Medical Center, in New Haven, Connecticut, followed by a plastic surgery residency at the University of Kansas Medical Center.^24^ His dual training in dentistry and medicine gave him a unique advantage in facial surgery, establishing him as a nationally recognized expert in the field.^24^

Kaye served as a clinical professor of plastic surgery and had a training program at the University of Florida College of Medicine, where he served as chief of plastic surgery at Baptist Medical Center in Jacksonville prior to his retirement in the 1990s. Outside of his medical career, Kaye was a true Renaissance man—he played multiple instruments and was once honored with an alto saxophone solo composed for him by Duke Ellington.^24^ He was also an accomplished marksman, serving in the US Coast Guard, and founded the Jacksonville chapter of the Châine des Rôtisseurs, an international gastronomic society dedicated to fine cuisine. Kaye’s legacy in aesthetic surgery extended beyond his clinical innovations. He was a respected mentor to many future surgeons, and he is widely regarded as a foundational figure in the development of modern aesthetic plastic surgery. He passed away on March 14, 2008, at the age of 80 in Jacksonville, Florida.^24^

### Dr Robert Singer (living)

Robert Singer has been in private practice in La Jolla, California since 1976 and has become internationally recognized for his contributions to aesthetic plastic surgery.^25^ He has served as President and Chair of the Board of Trustees of The Aesthetic Society (formerly the American Society for Aesthetic Plastic Surgery), as well as Chair of the Board of Trustees of the American Society of Plastic Surgeons.^25^ Singer’s efforts in advancing patient safety have been particularly impactful. He played a pivotal role in mandating that all plastic surgeons affiliated with major professional organizations operate exclusively in accredited or licensed facilities.^26^ As President of the American Association for Accreditation of Ambulatory Surgery Facilities (AAAASF), he helped establish the gold standard for outpatient surgical facility accreditation.^26^

Additionally, Singer founded the Hot Topic Taskforce for national plastic surgery organizations to evaluate and report on the safety and efficacy of emerging technologies and procedures.^27^ Over the past four decades, he has authored approximately 350 scientific publications and remains a highly sought-after speaker at surgical conferences both in the USA and internationally. He was also a founding member of The Aesthetic Surgery Education and Research Foundation, serving as its president from 2001 to 2004.^27^ In recognition of his outstanding contributions to the field, Singer received the Career Achievement Award from the Foundation in 2019, becoming only the ninth surgeon to be honored with this prestigious distinction. As of 2026, he still maintains an active practice.^28^

### Dr Sidney K. Wynn (1917–2003)

Sidney Wynn, from Milwaukee, Wisconsin, was a highly respected plastic surgeon recognized for his innovative contributions to reconstructive surgery. Among his most notable achievements was the development and popularization of the vertical scarred flap for transposition—a significant advancement in pediatric reconstructive techniques. This approach greatly improved outcomes in the correction of congenital deformities such as cleft lip and palate, particularly in very young patients, by minimizing visible scarring while maximizing functional restoration. Wynn’s pioneering work has been recognized in early plastic surgery literature and cited in foundational texts on reconstructive flap design and pediatric plastic surgery.^29^ His techniques introduced essential methods to the field, promoting safer, more effective, and aesthetically favorable outcomes in pediatric patients undergoing reconstructive procedures.

## JEWISH INNOVATORS IN BREAST AND RECONSTRUCTIVE SURGERY

### Dr Bernard Fisher (1918–2019)

Bernard Fisher made groundbreaking contributions to the treatment of early-stage breast cancer, fundamentally and profoundly changing the standard of care. He demonstrated that a simple lumpectomy combined with radiation therapy was just as effective, but significantly less disfiguring, than the traditional radical mastectomy, which had been the standard treatment for decades, leading to a major paradigm shift in breast cancer treatment.^30^

Born and raised in Pittsburgh, Pennsylvania, Fisher spent most of his academic and professional career at the University of Pittsburgh. Interestingly, his initial interests did not lie in cancer research. While at the University of Pennsylvania, he was mentored by Dr Isidor Ravdin, who encouraged him to attend a National Institutes of Health (NIH) conference on cancer clinical trials.^31^ This experience led Fisher to co-found and eventually lead the National Surgical Adjuvant Breast and Bowel Project (NSABP), through which he conducted his most influential studies. In addition to his work on lumpectomy and radiation therapy, Fisher also played a pivotal role in demonstrating the effectiveness of adjuvant chemotherapy for early-stage breast cancer patients—findings that have since been incorporated into modern clinical practice.^32^ Although not a plastic surgeon, his contributions to the field of plastic surgery were significant, as defining lumpectomy as an oncologically successful alternative to mastectomy led to subsequent innovations in breast reconstruction. His research laid the foundation for the multidisciplinary treatment approaches that remain the standard today.

However, Fisher’s career was not without controversy. Roughly a decade after his landmark lumpectomy study was published, allegations of data falsification and fraud emerged against one of his principal investigators. Although Fisher himself was not implicated, he was forced to resign from his positions at both the University of Pittsburgh and the NSABP.^33^ He was eventually fully vindicated, but the incident left a lasting mark on his reputation, and he remained a somewhat controversial figure.^33^ Despite the challenges he faced, Fisher’s pioneering work in women’s health continues to have a lasting impact. His contributions to breast cancer treatment remain widely recognized and continue to guide clinical practice worldwide.

### Dr Michael Scheflan (1945–2022)

Michael Scheflan made transformative contributions to breast reconstruction through his co-authorship of the landmark 1982 paper alongside Drs Carl Hartrampf Jr. and Paul Black, which introduced the transverse rectus abdominis myocutaneous (TRAM) flap for breast reconstruction.^34^,^35^ Born on June 4, 1945, Scheflan received his medical training in Switzerland before completing general surgery training at Albert Einstein (New York City), Mount Sinai (New York City), and Harvard University (Boston, Massachusetts). He later completed his plastic surgery training at Emory University in Atlanta, Georgia, under the mentorship of Dr Foad Nahai beginning in 1978.^36^

Board-certified by both the American Board of Surgery and the American Board of Plastic Surgery—and holding equivalent certifications in Israel^34^—Scheflan’s career spanned continents. He served as an assistant professor of plastic surgery at Virginia Commonwealth University and as chief physician at RoKach Medical Center in Tel Aviv.^36^ Fluent in German, French, English, Hebrew, and Italian, Scheflan forged meaningful global connections and contributed extensively to the international plastic surgery community. He also served as president of the Israel Society of Plastic Surgeons and was honored with numerous prestigious awards, including the James Barrett Brown Award, the Carl Moyer Award, the Raymond Villan Award, and the Maliniac Lecture Award. Scheflan passed away unexpectedly on February 12, 2022, at the age of 76, leaving a significant legacy in international plastic surgery.^36^

### Dr Jack Penn (1909–1996)

Jack Penn, born and trained in South Africa, is widely regarded as one of the forefathers of plastic surgery in Israel. He received his medical degree from the University of Witwatersrand in Johannesburg and completed his surgical training in the United Kingdom and Mayo Clinic in Rochester, Michigan. During Israel’s War of Independence in 1948, Penn volunteered to care for wounded soldiers under austere conditions, helping to establish foundational reconstructive surgical services. His leadership was instrumental in the development of organized plastic surgery training and practice in Israel, and he later played a central role in founding the Israeli Society of Plastic and Aesthetic Surgery. He also founded the South African Association of Plastic and Reconstructive Surgery and served as its first president.^20^

### Dr Jack Fisher (living)

Jack Fisher is a prominent figure in aesthetic and reconstructive breast surgery. He earned his medical degree from Emory University School of Medicine in Atlanta, Georgia, and completed his plastic surgery residency at Emory University Affiliated Hospitals.^37^ Following residency, he joined the Mayo Clinic in Rochester, Minnesota, serving as an attending plastic surgeon from 1981 to 1986, with a specialty focus on aesthetic and reconstructive breast procedures.^37^ In 1986, Fisher relocated to Nashville, Tennessee, where he established a private practice centered on breast surgery, especially revisionary and secondary procedures. He also served as Associate Clinical Professor of Plastic Surgery at Vanderbilt University Medical Center in Nashville, Tennessee and was the Immediate Past President of The American Society for Aesthetic Plastic Surgery.^37^

Beyond breast surgery, Fisher has been internationally recognized for his work in hair transplantation, having trained under Dr Carlos Uebel in Brazil—one of the pioneers of the small graft megasession.^37^ Fisher has become renowned for his expertise in correcting failed hair transplants and has developed educational resources for training physicians in this area. In 1998, he performed the world’s first hair transplant broadcast live over the internet and has been consistently listed in *Woodward/ White’s Best Doctors in America* since 1997.^37^ He has been active in several leading medical organizations, including the International Society of Hair Restoration Surgery, the American Society for Aesthetic Plastic Surgery, and the American Society of Plastic Surgeons.^38^

### Dr Berish Strauch (1933–2023)

Berish Strauch was a trailblazer in microsurgery, whose innovations fundamentally reshaped reconstructive plastic surgery. Born on September 19, 1933, in the South Bronx in New York to immigrant parents, Strauch graduated from Bronx High School of Science and Columbia University College of Physicians and Surgeons in New York, after which he completed his general surgery residency at Montefiore Medical Center, also in New York.^39^ After serving as a captain in the US Army, he pursued plastic surgery training at Stanford University in California under the mentorship of Dr Robert Chase, where he began his groundbreaking work in microsurgery. In 1978, Strauch became chief of the plastic surgery division at Montefiore, later elevating it to departmental status in 1987, and served as chairman until his retirement in 2007. Among his many surgical milestones was one of the earliest toe-to-thumb transplants, performed in 1976 on a New York City firefighter, which showcased the potential of microsurgical reconstruction.^39^

Strauch also invented the first inflatable penile prosthesis and developed techniques for removing excess skin in patients following bariatric surgery.^40^ His contributions to urology included the invention and patenting of the Strauch Clamp, used in vasectomy reversals and other procedures.^41^ Strauch co-authored the *Encyclopedia of Body Sculpting After Massive Weight Loss* with Charles Herman in 2010. Also, he co-edited *Grabb’s Encyclopedia of Flaps* with Luis O. Vasconez.^41^ He was honored with the Distinguished Fellowship Award from the American Association of Plastic Surgeons in 2023.^36^ He passed away on December 4, 2023, at the age of 90, leaving behind a legacy of innovation and a generation of surgeons trained in microsurgical techniques.^40^

## CONTRIBUTORS TO THE EVOLUTION OF EDUCATION AND GLOBAL COLLABORATION

### Dr Robert Malcolm Goldwyn (1930–2010)

Robert Malcolm Goldwyn, born in Worcester, Massachusetts in 1930, emerged as one of the most influential figures in plastic surgery literature and education. He graduated with Phi Beta Kappa honors from Harvard College of Cambridge, Massachusetts, in 1952 and earned his medical degree from Harvard Medical School in 1956.^41^ Goldwyn completed his general surgery training as a Harvey Cushing Fellow at Peter Bent Brigham Hospital in Boston, Massachusetts, and underwent plastic surgery training at the University of Pittsburgh Medical Center under Dr William White. He later served as Chief of the Division of Plastic Surgery at Beth Israel Hospital in Boston, Massachusetts, from 1972 to 1996 and was Clinical Professor of Surgery at Harvard Medical School.^41^

Goldwyn’s most enduring legacy was his 25-year tenure as editor-in-chief of *Plastic and Reconstructive Surgery* (1979–2004), during which he elevated the journal to become the leading publication in the field.^41^ Under his leadership, the journal expanded in scope and circulation, introduced colorized images, and launched online publication in 1999. In 1972, he founded the National Archives of Plastic Surgery at the Francis A. Countway Library of Medicine in Boston, preserving the specialty’s historical record for future generations. A prolific author, Goldwyn published more than 300 articles and edited numerous influential texts, including *The Unfavorable Results in Plastic Surgery: Avoidance and Treatment*.^42^ Beyond surgery, he was deeply engaged in medical ethics, world peace, and social advocacy. He was a founding member of Physicians for Social Responsibility and wrote passionately against chemical and biological warfare. Goldwyn passed away on March 23, 2010, at the age of 79, following a 16-year battle with prostate cancer.^41^

### Dr Irving Goldman (1898–1975)

Irving Golding was a highly influential American plastic surgeon whose career was defined by his contributions to the education and training of plastic surgeons, mainly in New York City at Mount Sinai Hospital. Golding played a pivotal role in establishing and advancing formal residency education in plastic surgery, helping to shape standardized training pathways during a formative period in the specialty’s development. Through his academic leadership, teaching, and mentorship, he contributed to the professionalization of plastic surgery and influenced generations of surgeons who went on to academic and clinical leadership roles.^43^

### Dr Samuel Fomon (1889–1971)

Samuel Fomon, from Chicago, Illinois, represents a unique figure in the history of plastic surgery as a scholar and educator rather than a practicing clinician. Fluent in German and deeply engaged with European surgical literature, Fomon studied under Jacques Joseph and authored *Surgery of Injury and Plastic Repair*, one of the earliest comprehensive English-language texts synthesizing reconstructive principles. Through his anatomical teaching courses in Boston and New York, he profoundly influenced generations of surgeons despite never maintaining an independent surgical practice.^44^

### Dr Gaston Schwarz (1938–2022)

Gaston Schwarz was a trailblazer in plastic surgery education and international cooperation. Born on January 24, 1938, he devoted more than five decades to the field as both a surgeon and academic.^45^ In 1983, Schwarz revolutionized surgical education by introducing live surgery teaching to plastic surgery conferences—an innovation that soon became standard practice. He was among the first to open a private operative facility in Montreal, Canada and played a key role in establishing safety protocols and certification standards for private surgical clinics.^45^

For 40 years, Schwarz’s private clinic served as a cornerstone of aesthetic surgery training for McGill University (Montreal, Canada) plastic surgery residents, offering generations of trainees unparalleled clinical experience.^45^ He served as President of the Canadian Society of Aesthetic Plastic Surgeons and received its Lifetime Achievement Award in recognition of his leadership and influence in the field.^46^ An active humanitarian, Schwarz spearheaded efforts to equip underserved hospitals in Peru and Bolivia with modern medical equipment from Canada—work that earned him national honors from both countries. In 1985, he established the Montreal Plastic and Aesthetic Surgery Center, which later received exemplary accreditation from Accreditation Canada.^46^ Schwarz passed away on January 20, 2022, just four days shy of his eighty-fourth birthday. To honor his legacy, McGill University established the Dr Gaston Schwarz Memorial Fund in Plastic Surgery, supporting ongoing advancements in surgical education and research.^45^

## JEWISH INNOVATORS IN COSMETIC SURGERY AND PROFESSIONAL SOCIETIES

### Dr Richard Webster (1918–1994)

In the 1980s, Richard Webster, widely recognized as “the father of cosmetic surgery,” played a key role in the establishment of the American Academy of Cosmetic Surgery (AACS). Born in Baltimore, Maryland, Webster graduated from both Harvard University and Harvard Medical School. Among his many contributions to the field were advancements in the treatment of full thickness burn wounds and the surgical reconstruction of the *labium inferius oris* (lower lip). Webster improved burn care by emphasizing the importance of early surgical intervention and modernized skin grafting techniques to minimize infection and promote healing.^47^ In facial reconstruction, he introduced the use of innervated, muscle-bearing flaps to enhance functional outcomes in lower lip repair—an advancement on a technique originally developed by Christian Bernard in the late 1800s.^48^ This innovation became known as the “Bernard–Webster flap.”^49^

Webster had a particular passion for cosmetic surgery. He and several colleagues recognized that the field was not receiving adequate recognition within general plastic surgery. Motivated by this, they helped establish the American Academy of Cosmetic Surgery, which now boasts over 1,600 members and serves as a leader in providing educational resources across cosmetic disciplines. Beyond surgical innovation, Webster was deeply committed to education and global collaboration. He lectured and visited more than 30 institutions worldwide, promoting knowledge exchange and the growth of aesthetic surgery.^50^ His philosophy and dedication have inspired countless surgeons and continue to influence the field today.

### Dr Simon Fredricks (1926–2018)

In Texas, Simon Fredricks played a similarly pivotal role in advancing the field of aesthetic surgery. Fredricks collaborated with Webster and a small group of like-minded plastic surgeons to form what would become the American Society for Aesthetic Plastic Surgery (ASAPS). Driven by a passion for refining cosmetic techniques and sharing ideas, they laid the groundwork for a society focused solely on aesthetic advancements.^51^

Fredricks was known for his patient-centered approach, often favoring more conservative procedures. He cautioned colleagues against the risks of overly aggressive facelifts, advocating for safer, more natural outcomes.^51^ In 1967, Fredricks co-founded the Society of Aesthetic Surgeons—a precursor to ASAPS—alongside Dr John Lewis. According to accounts, the two famously sketched their vision for the society and a list of prospective members on a napkin in a bar in Italy.^52^ Fredrick’s contributions were instrumental in shaping the educational and clinical focus of aesthetic surgery. Drs Stanley Klatsky from Baltimore and Foad Nahai, originally from Iran and later Atlanta, Georgia, further supported ASAPS’s mission by serving as editors of the society’s journal, advancing academic scholarship and outreach in the field, which was begun by Dr Robert Bernard, who trained at New York University and practiced in White Plains, New York.^52^

## BRINGING ANGIOGENESIS TO CANCER TREATMENT

### Dr Judah Folkman (1933–2008)

Judah Folkman, born in Cleveland, Ohio, was the son of a rabbi and developed an interest in medicine at a young age. By the age of 19, he was already enrolled in medical school. While still a student, he invented one of the first implantable pacemakers of its kind.^53^ In the 1970s, while investigating the process of angiogenesis, Folkman theorized that targeting this biological process could significantly inhibit tumor growth—and potentially eliminate tumors altogether.^54^

His groundbreaking theory gained widespread attention in the 1980s when *The New York Times* reported on two drugs he had developed that successfully eradicated tumors in mice.^55^ Although cancer has proven significantly more difficult to treat in humans than in animal models, Folkman’s work represented a major breakthrough and inspired researchers worldwide to pursue alternative cancer therapies. His theory that tumor growth depends on angiogenesis remains widely accepted today.^56^ Notably, he also foresaw the clinical use of anti-angiogenic antibodies to control tumor progression.^57^

Folkman’s brilliance was recognized early in his career: at just 34 years old, he was appointed surgeon-in-chief at Children’s Hospital Boston, Massachusetts, only 15 years after his acceptance to Harvard Medical School. Over the course of his career, he has authored approximately 400 scientific papers and received numerous awards and accolades. Among his most significant contributions was a 1990 thesis that reaffirmed his life’s work, asserting that tumors cannot grow unless preceded by capillary development.^58^ To support this, he referenced earlier research using an *in vivo* rabbit model in which his team implanted tumor cells into the corneal stroma—an area normally devoid of capillaries. The experiment demonstrated a dramatic increase in neovascularization, providing compelling evidence of angiogenesis as a prerequisite for tumor growth.^59^ Folkman’s pioneering work fundamentally transformed the scientific understanding of tumor biology and laid the groundwork for modern anti-angiogenic therapies.

## CONTEMPORARY LEADERS IN AESTHETIC AND RECONSTRUCTIVE PLASTIC SURGERY

### Dr Scott L. Spear (1948–2017)

Scott L. Spear was a leading figure in aesthetic and reconstructive breast surgery, earning his medical degree from the University of Chicago and completing his general surgery residency at Harvard Medical School, followed by his plastic surgery residency at the University of Miami and a craniofacial fellowship at L’Hospital Enfants Malades in Paris in 1980.^60^ Spear served as founding Chairman of the Department of Plastic Surgery at Georgetown University Hospital in Washington, D.C., and was instrumental in developing the Washington area training program in plastic surgery. He served as President of the American Society of Plastic Surgeons from 2004 to 2005 and was widely recognized as an icon in plastic surgery.^61^

Spear’s most significant contribution was his pioneering work in nipple-sparing mastectomy, heretofore not done, which revolutionized breast reconstruction surgery.^62^ He was instrumental in helping to end the longstanding US Food and Drug Administration restrictions on the use of silicone gel breast implants and provided creative solutions for improved outcomes in primary and secondary breast implant surgery.^62^ Over his career, Spear published more than 250 papers and chapters, participated in over 900 invited lectures, and authored three editions of the seminal textbook *Surgery of the Breast: Principles and Art*.^60^ He served as breast section editor for the *Plastic and Reconstructive Surgery Journal* and was frequently featured in major media outlets. Spear was posthumously awarded the 2018 Career Achievement Award by the Aesthetic Surgery Education and Research Foundation, and was further honored by the American Society of Plastic Surgeons with a posthumous award at its 2025 annual meeting in New Orleans.^62^ He passed away peacefully in his sleep on March 16, 2017, aged 68.^60^

### Dr Alan Matarasso (1953—)

Alan Matarasso is a native of Westchester County, New York. He completed his general surgery and plastic surgery training at Albert Einstein College of Medicine–Montefiore Hospital under Berish Strauch and pursued a fellowship in aesthetic surgery at Manhattan Eye, Ear and Throat Hospital, New York, under Thomas D. Rees at a time when cosmetic surgery was gaining recognition as a discipline within plastic surgery.^63^ Matarasso devoted his entire career to aesthetic plastic surgery and has served as Professor of Plastic Surgery at Albert Einstein and Clinical Professor of Surgery at Northwell Health System/Hofstra University, Zucker School of Medicine in New York.^64^ He served as President of the American Society of Plastic Surgeons from 2018 to 2019 and currently serves as President of the Plastic Surgery Foundation as of 2025.^65^

He pioneered the foundation of classification and treatment in abdominoplasty surgery, facial contouring, and reshaping. Matarasso serves as assistant national secretary (USA) to the International Society of Aesthetic Plastic Surgery (ISAPS) and as the US delegate to the International Confederation of Plastic Surgery Societies (ICOPLAST).^65^ He previously served as the president of The Rhinoplasty Society in 2009 and the New York Regional Society of Plastic Surgeons in 2006. His dedication to the field has been recognized through numerous awards and his inclusion in multiple “Best Doctors” lists, including one of two plastic surgeons named in the top 10 of all categories by *Newsweek* magazine.^65^

Matarasso has authored more than 1,000 peer-reviewed journal articles, publications, book chapters, monographs, and reviews, making him one of the most prolific writers in plastic surgery. He is the co-editor of the most recent Neligan textbook section on cosmetic surgery with Peter Rubin and the forthcoming Expert Techniques in Aesthetic Surgery. He has delivered more than 700 presentations, lectures, and panels to share his expertise on aesthetic plastic surgery and is frequently quoted in national newspapers and magazines.^65^

### Dr Vladimir Mitz (1943–)

Vladimir Mitz is a French plastic surgeon and anatomist whose work has fundamentally reshaped modern understanding of facial anatomy and rejuvenation surgery. He achieved international recognition through this seminal description of the superficial musculoaponeurotic system (SMAS), which clarified the structural continuity between the platysma, facial musculature, and overlying soft tissues and provided a durable anatomical basis for modern facelift techniques.^66^ By shifting surgical focus from skin-only excision to manipulation of deeper fascial layers, Mitz’s work is enabling more natural, durable, and anatomically sound approaches to rhytidectomy. His anatomical insights continue to underpin contemporary facial rejuvenation surgery and remain foundational to both aesthetic and reconstructive procedures involving the aging face.

### Dr James M. Stuzin (1952–)

James M. Stuzin graduated from the University of Florida College of Medicine in 1978 and completed his plastic surgery residency at New York University Grossman School of Medicine from 1984 to 1986.^67^ He also completed fellowship training under Drs S. A. Wolfe and Henry Kawamoto. He has established himself as one of the foremost experts in aesthetic plastic surgery, with over 40 years of experience in facial rejuvenation. Stuzin’s groundbreaking work on facial anatomy contributed to modern facelift techniques, with his early anatomical studies becoming classics that are frequently cited by researchers and surgeons worldwide. While succeeding Dr Howard Gordon in practice and working with Dr Thomas J. Baker, Stuzin developed the extended superficial musculoaponeurotic system (SMAS) procedure, a widely utilized technique in facelifts that revolutionized aesthetic approaches to facial rejuvenation.^68^ He has served as Chairman of the Baker Gordon Educational Symposium on Cosmetic Surgery, the oldest symposium of cosmetic surgery in the world.^69^

Stuzin served as President of The Aesthetic Society from 2007 to 2008 and received the 2023 Aesthetic Surgery Education and Research Foundation Career Achievement Award.^69^ His research on facial fat compartments has provided an anatomical roadmap for facial rejuvenation procedures and explained regional facial aging patterns.^70^ In addition to his academic and clinical contributions, Stuzin served as co-editor of *Plastic and Reconstructive Surgery*, reflecting his leadership in shaping contemporary plastic surgery literature.^67^ Stuzin has authored several seminal textbooks including *Surgical Rejuvenation of the Face* and *Facial Skin Resurfacing*, and has published over 100 heavily cited articles. He was listed in America’s Best Plastic Surgeon by *Newsweek* magazine in both 2021 and 2022 in the facelift category. His live surgery demonstrations are among the most widely viewed videos by plastic surgeons globally and are archived on The Plastic and Reconstructive Surgery Journal website.^67^

### Dr Bryan Mendelson (living)

Bryan Mendelson is a world-renowned Australian plastic surgeon and anatomist whose work has profoundly shaped contemporary understanding of facial aging and aesthetic facial surgery. Trained at the Mayo Clinic of Rochester, Minnesota, in 1971, he practiced for more than four decades at the forefront of facial rejuvenation surgery and is internationally recognized for his original research on facial anatomy, particularly facial spaces and retaining ligaments.^71^ His anatomical discoveries, published in numerous peer-reviewed scientific journals and incorporated into the online edition of *Gray’s Anatomy*, enabled the development of facial rejuvenation techniques that work in harmony with underlying anatomy and prioritize natural outcomes.^72^,^73^

Following four decades of operative practice, Mendelson no longer performs surgery but continues to contribute as a Facial Aesthetic Surgery Consultant. In addition to his academic and clinical achievements, he has served as President of the International Society of Aesthetic Plastic Surgery (ISAPS) and has supported educational access for migrant children through a scholarship established in honor of his father.^74^

### Dr Harvey Zarem (1932–2015)

Harvey Zarem was born on February 13, 1932, in Savannah, Georgia, and became widely regarded as the “Dean of Plastic Surgery.”^75^ He graduated from Yale University in New Haven, Connecticut, and earned his medical degree from Columbia University College of Physicians and Surgeons in New York City in 1957.^76^ Zarem completed his general surgery training as Chief Resident at Harvard Medical School and his plastic surgery residency at Johns Hopkins Hospital in Baltimore, Maryland.^75^ He served as Professor and Chief of Plastic and Reconstructive Surgery at the University of Chicago for seven years before holding the same position at University of California, Los Angeles Medical Center and School of Medicine for 14 years.^75^

Zarem pioneered numerous techniques that became commonplace in plastic surgery, including liposuction and reconstructive post-cancer surgery of the breast. He produced seminal treatises on eyelid surgery and treatments of hemangiomas in infants and children.^77^ Zarem trained over 50 leading plastic surgeons in the USA and elsewhere throughout his career. He appeared on multiple episodes of the American TV show, “Extreme Makeover” and served as a medical consultant on the Tom Cruise film *Vanilla Sky*.^78^ Zarem ran a private practice in Santa Monica, California, before returning to his hometown of Savannah in 2011. He passed away on November 1, 2015, at age 83 at his home in Savannah.^75^

### Dr Jeffrey E. Janis (living)

Jeffrey E. Janis, MD, FACS, is a leading contemporary figure in academic and clinical plastic surgery. He serves as Professor and Executive Vice Chairman of Plastic Surgery at The Ohio State University Wexner Medical Center.^79^ Renowned for his expertise in complex craniofacial and reconstructive surgery, Janis’s research has focused on abdominal wall reconstruction, migraine surgery, pain management, and resident education. He has co-edited multiple highly influential supplements in the *Plastic and Reconstructive Surgery* journal on topics including wound healing, acellular dermal matrices, pain management, and abdominal wall reconstruction.^80^ Furthermore, Janis was part of the surgical team that performed the first full face transplant in the USA, marking a watershed moment in craniofacial surgery and transplantation.^81^ He has served as President of the American Society of Plastic Surgeons (ASPS), chairing numerous national committees and delivering over 725 lectures worldwide.^81^ Through nearly 100 peer-reviewed articles, 70 book chapters, and several books, Janis has advanced the fields of reconstructive and aesthetic plastic surgery and contributed greatly to multidisciplinary surgical education, and is the current editor of *Plastic Surgery Global Open*.

## LEADERSHIP AND INNOVATION IN MAXILLOFACIAL SURGERY AND THE ASMS

### Dr Arthur Ship (1928–2017)

Arthur Ship’s career reflects both technical mastery and a lifelong dedication to advancing the art and science of plastic surgery. Born in 1928 to Jewish immigrants from Russia, Ship’s early intellectual and artistic talents took him from the Bronx to The Julliard School of Music in New York City, and ultimately to Harvard, where he earned both his DMD (1952) and MD (1954) within six years.^82^ Board-certified in plastic surgery in 1963, Ship established a private practice in New York City, while maintaining an enduring affiliation with Montefiore Hospital nearby. There, he became known for his engaging, hands-on teaching style, which fostered deep learning among residents. With his unique background in dentistry and maxillofacial surgery, Ship developed specialized teaching courses, such as maxillofacial modeling and standardized patient photography, cementing his role as an innovator in surgical education.^82^

Beyond the operating room, Ship was a leader and builder of professional institutions. He served as president of the American Society of Maxillofacial Surgeons (1983–1984), and he was a founding member of the Board of Trustees of the AAAASF. Also, Ship held leadership roles at Montefiore, including president of its Medical Staff and Alumni Association. His professional contributions spanned research, peer-reviewed publications, book chapters, and visiting professorships, with a particular interest in historical perspectives on surgical practice. Ship’s commitment to service extended internationally, notably when he traveled to Israel during the 1973 Yom Kippur War to care for the wounded.^82^,^83^ By combining innovation in maxillofacial techniques, dedication to surgical safety, and a strong ethic of mentorship, Ship not only advanced plastic surgery as a discipline but also inspired generations of surgeons who continue to benefit from his teachings. He was instrumental in the initiation and development of the ASMS Basic Maxillofacial Course.

### Dr John H. Garlock (1896–1965)

John H. Garlock was a foundational figure in the institutional development of plastic surgery in the USA and played a major role in establishing plastic surgery at Mount Sinai Hospital in New York City. Garlock was instrumental in building early academic and clinical plastic surgery programs and was a founding member of the American Board of Plastic Surgery (ABPS), contributing to the formalization of training standards and board certification in the specialty.^84^,^85^ His leadership helped legitimize plastic surgery as a distinct surgical discipline within American academic medicine and laid groundwork for subsequent generations of reconstructive and aesthetic surgeons.

### Dr Casper Morley Epsteen (1902–1995)

Casper Morley Epsteen was a physician and educator who played a key role in establishing maxillofacial surgery as a recognized surgical specialty. After completing both medical and dental training,^86^ he combined these skill sets to specialize in treating complex disorders of the face and jaws—areas that required expertise across medicine, dentistry, and reconstructive surgery. He practiced for nearly seventy years in Chicago while also teaching as Clinical Professor of Maxillofacial and Plastic Surgery at Chicago Medical School, later becoming Professor Emeritus.^86^

Beyond his clinical expertise, Epsteen was a founding member of the American Society of Maxillofacial Surgeons and served as its president in 1960, helping to create professional structures that shaped the field. His research on salivary gland disorders earned him recognition from the American Society of Plastic and Reconstructive Surgeons in 1953, and he was honored by leading Chicago hospitals for his teaching and service. His reputation extended internationally, culminating in being named Guest of Honor at the First International Congress of Maxillofacial Surgeons in Venice in 1971.^86^ As a Jewish physician working in an era of professional barriers, his leadership in founding surgical societies, advancing academic teaching, and bridging plastic and dental surgery underscored both his resilience and his enduring contributions to the development of modern reconstructive surgery.

### Dr Joseph S. Gruss (1945–2019)

Joseph S. Gruss was a foundational figure in craniomaxillofacial surgery, particularly noted for pioneering the use of rigid internal fixation and primary bone grafting in the treatment of complex facial fractures. First at Sunnybrook Hospital in Toronto, and later at the University of Washington in Seattle, he transformed the standard of care for craniofacial trauma.^87^ His dedication to training and mentorship—guiding more than 100 residents and 60 craniofacial surgical fellows—stands as a testament to his lasting influence on the specialty, further reflected in the honors he received, including the American Association of Plastic Surgeons (AAPS) Distinguished Fellow Award, the ASMS Lifetime Achievement Award, and an Honorary Fellowship from the Royal Australasian College of Surgeons.^88^

## EDUCATIONAL LEADERSHIP WITHIN THE ASMS

Dr Jeffrey L. Marsh contributed to the evolution of craniomaxillofacial surgery through early adoption of 3-D imaging techniques that revolutionized diagnosis and preoperative planning for congenital deformities.^89^

Meanwhile, Dr Irving Polayes was instrumental in founding and expanding the ASMS Basic Maxillofacial Course—the first long-running, multi-day hands-on training series for residents—which emphasized not only surgical techniques but also underlying head and neck anatomy, embryology, dental anatomy, and radiology.^90^ He was also closely connected to the launch of *Plastic Surgery News* in 1980, published under the joint auspices of ASMS and American Society of Plastic Surgeons (ASPS), reflecting his leadership during a formative period for the society’s communication and professional identity.

Other key contributors to ASMS’s educational and scientific mission include Drs Raymond Kaufman, Samuel Shatkin, and John Jacobs—leaders who helped establish the society’s pedagogical structure and research foundations, ensuring that trainees received rigorous preparation for maxillofacial and craniofacial practice.^90^

## SHAPING THE MODERN UNDERSTANDING OF CRANIOFACIAL SURGERY

### Dr Janusz Bardach (1919–2002)

Janusz Bardach was a pioneering craniofacial surgeon whose work significantly advanced modern approaches to cleft lip and palate repair, particularly with respect to preservation of maxillary growth. Born in Odessa, Ukraine, in 1919 and raised in Poland, Bardach later trained in reconstructive maxillofacial surgery at the Moscow Medical Stomatological Institute. He developed innovative techniques in cleft palate repair, most notably the two-flap palatoplasty, which became widely adopted in clinical practice.^91^ After emigrating to the USA in 1972, Bardach was appointed Chief of the Division of Plastic Surgery at the University of Iowa, where his experimental and clinical studies demonstrated that minimizing surgical trauma to the developing maxilla resulted in more normal facial growth—findings that continue to influence contemporary standards in pediatric craniofacial surgery.^92^

### Dr Daniel Marchac (1936–2012)

Daniel Marchac served as Professor of Plastic Surgery at the Collège de Médecine des Hôpitaux de Paris and was a key architect of contemporary craniofacial surgery. His pioneering work in the early surgical management of craniosynostosis—particularly frontocranial remodeling in infancy—established foundational techniques still referenced in specialist practice today.^93^ Alongside Dominique Renier, Marchac co-authored a valuable clinical text on craniofacial surgery and helped shape the discipline through formative publications.^94^ He also co-founded the International Society of Craniofacial Surgery, which continues to lead internationally as the forum for craniofacial surgery.^95^ Beyond craniosynostosis, his surgical innovations extended into areas such as basal cell carcinoma treatment nasal reconstruction, endoscopic forehead lifts, and breast surgery, showcasing his versatile impact on plastic surgery education and clinical evolution.

### Dr Michael Lewin (c. 1910–1991)

Michael Lewin, from Warsaw, Poland, and educated at the University of Zurich, left a lasting legacy in craniofacial medicine through both his clinical leadership and his educational influence. He served for 18 years as the Founder and Chief of Plastic Surgery at Montefiore Medical Center and, subsequently, as Director of Plastic Surgery at the Albert Einstein College of Medicine, both in New York City.^96^ Lewin also established and directed The Center for Craniofacial Disorders at the same institution, marking a key milestone in organized specialty care. In 1976, he developed a workshop on basic maxillofacial surgical techniques paired with a jaw symposium in Philadelphia, thereby contributing notably to the dissemination of expertise in jaw deformity correction. That same year, he collaborated with Drs Rankow and Casey to organize the first successful Maxillofacial Basic Workshop in Chicago, further solidifying his reputation as a pioneering educator.^97^ His prolific authorship and presentations at international forums reinforced his influence as both an innovator and a teacher within the field.

## EDUCATORS, HUMANITARIANS, AND VISIONARIES BEYOND THE OPERATING ROOM

### Dr Melvin “Mel” Spira (1925–2020)

Melvin “Mel” Spira exemplified the ideals of lifelong education, leadership, and global service in plastic surgery. He graduated from Northwestern University Dental School in Chicago, Illinois, in 1947, then the Medical College of Georgia in 1956. This was followed by general surgery training at Duke University in North Carolina and plastic surgery residency at Baylor College of Medicine in Houston, Texas, where he became Chief of Plastic Surgery in 1976, serving nearly two decades in that role.^98^ Renowned as a prolific educator, he authored over 200 peer-reviewed articles, contributed more than 35 book chapters, and served as a visiting professor at 50 institutions globally.^99^

Outside academia, Spira was deeply committed to humanitarian medicine. He volunteered on dozens of overseas surgical missions and continued to provide cleft lip and palate surgery late in life, including his final trip to Vietnam at age 89.^100^ As a mentor, he infused surgical training with kindness and curiosity, and his legacy endures through the Melvin Spira, MD, Endowed Chair in Plastic Surgery at Baylor—an enduring tribute to his vision as both a teacher and a philanthropist.^100^

### Dr Bernard G. Sarnat (1912–2011)

Bernard G. Sarnat was a pioneering surgeon, basic science researcher, and philanthropist whose work profoundly shaped the understanding of craniofacial development. Born and raised in Chicago, Sarnat earned his BS and MD from the University of Chicago and his MS and DDS from the University of Illinois in 1940. His groundbreaking research on the “functional matrix” theory—that soft tissues dictate bone growth and form—advanced the scientific underpinnings of facial development and malformation management.^101^ In 2010, he summarized decades of scholarship in his seminal book *Craniofacial Biology and Craniofacial Surgery*.^102^ He authored over 220 peer-reviewed articles, lectured widely, and was a founding member of the Plastic Surgery Research Council.^103^ An innovator in early distance learning, Sarnat established a telephone-based lecture network that broadcast dental education to over 12,000 listeners across 260 cities long before the internet made virtual learning ubiquitous.^104^ Sarnat’s legacy endures through an excellence-in-grant-writing award in his name, recognizing researchers in craniofacial growth and development.

### Dr Arthur J. Barsky (1898–1982)

Arthur J. Barsky Jr. emerged as a visionary in global reconstructive plastic surgery, well beyond the confines of the operating room. As Chief of Plastic Surgery at Mount Sinai Hospital in New York City, he orchestrated the landmark “Hiroshima Maidens” project in 1955, in which 25 young women severely disfigured by the atomic bombing in Japan were brought to New York for reconstructive surgery. Barsky led the surgical team *pro bono*, with operations performed in donated hospital beds and patients housed in Quaker homes, symbolizing compassion transcending political tensions.^105^ Over the course of the women’s convalescence, the team performed approximately 138 surgeries and taught visiting Japanese physicians, thus strengthening surgical knowledge across nations.

Beyond this landmark effort, Barsky championed global humanitarian initiatives, co-founding Children’s Medical Relief International and establishing a pediatric plastic surgery unit in Saigon during the Vietnam War. This was aimed at training local surgeons and expanding access to reconstructive care amid conflict.^105^ His influence extended into surgical education worldwide: Christopher Hughes et al. described Barsky as “arguably one of the most influential forefathers of global plastic surgery,”^106^ emphasizing the originality, scale, and enduring global impact of his work.

### Dr Seth R. Thaller (1949–)

Seth R. Thaller has played a significant role in advancing craniofacial and reconstructive plastic surgery through clinical leadership, scholarship, and education. As Professor of Surgery and former Chief of the Division of Plastic Surgery at the University of Miami Miller School of Medicine, Thaller helped build and lead a comprehensive academic plastic surgery program with a strong emphasis on craniofacial reconstruction and resident education. He is the author and editor of numerous influential textbooks and reference works in plastic and craniofacial surgery and has published extensively in the peer-reviewed literature, contributing to the dissemination of operative principles, outcomes research, and educational standards. In addition to his scholarly output, Thaller has held national leadership roles within major professional societies and has been widely recognized for his commitment to training future generations of plastic surgeons through mentorship, curriculum development, and continuing medical education.^107^,^108^

## CONCLUSION

This historical review is not intended to be exhaustive, but rather to present representative examples of Jewish physicians whose documented contributions substantially shaped the development, practice, and conceptual foundations of plastic and reconstructive surgery across multiple eras and disciplines. Additional examples of Jewish plastic surgeons who have held leadership positions in academic institutions and professional organizations are summarized in Table 1.

**Table 1:** Jewish Plastic Surgeons in Academic and Institutional Leadership in the USA.

Name	Institution	Leadership Role
Mimis Cohen	University of Illinois Health	Director, Craniofacial Center Chief, Division of Plastic, Reconstructive, and Cosmetic Surgery

Evan Garfein	Albert Einstein College of Medicine (New York City)	Chief, Plastic and Reconstructive Surgery

William Hoffman	University of California, San Francisco	Former Chief, Division of Plastic & Reconstructive Surgery
Jeff Marcus	Duke University (North Carolina)	Chief, Division of Plastic, Maxillofacial, and Oral Surgery
Bruce Mast	University of Florida	Division Chief

Babak Mehrara	Memorial Sloan Kettering Cancer Center (New York City)	Chief, Plastic and Reconstructive Surgical Service
Justin Sachs	Barnes-Jewish Hospital (St. Louis, Missouri)	Chief, Division of Plastic & Reconstructive Surgery
Sumner Slavin	Beth Israel–Deaconess Medical Center (Boston, Massachusetts)	Co-Director, Aesthetic Surgery Fellowship Program Former Chief, Plastic Surgery
Samuel Stal	Baylor College of Medicine (Houston, Texas)	Former Chief, Plastic Surgery

Peter Taub	Mount Sinai Hospital (New York City)	System Chief, Division of Plastic and Reconstructive Surgery
Seth R. Thaller	University of Miami (Florida)	Former Chief, Division of Plastic, Aesthetic, and Reconstructive Surgery

This table is representative of Jewish plastic surgeons who have held leadership positions in academic institutions and professional organizations. It was compiled by the authors based on publicly available institutional and professional society information. All positions are current unless indicated otherwise.

The contributions of Jewish surgeons and physicians have played and will continue to play a pivotal role in the advancement of plastic and reconstructive surgery. Their innovative surgical techniques, dedication to ethical patient care, and commitment to research and teaching have helped establish plastic surgery as a discipline focused on restoring form, function, and quality of life. Moreover, the foundational work of these early pioneers paved the way for the field’s continued evolution, with subsequent generations of Jewish plastic surgeons carrying forward this legacy and making significant contributions to modern surgical practice.

## Abbreviations

AAAASF: American Association for Accreditation of Ambulatory Surgery Facilities
AAPS: American Association of Plastic Surgeons
ASAPS: American Society for Aesthetic Plastic Surgery
ASMS: American Society of Maxillofacial Surgeons
ASPS: American Society of Plastic Surgeons
NIH: National Institute of Health
NSABP: National Surgical Adjuvant Breast and Bowel Project
